# PI signal transduction and ubiquitination respond to dehydration stress in the red seaweed *Gloiopeltis furcata* under successive tidal cycles

**DOI:** 10.1186/s12870-019-2125-z

**Published:** 2019-11-27

**Authors:** Shun Liu, Zi-Min Hu, Quansheng Zhang, Xiaoqi Yang, Alan T. Critchley, Delin Duan

**Affiliations:** 1 Key Laboratory of Experimental Marine Biology, Center for Ocean Mega-Science, Institute of Oceanology, Chinese Academy of Sciences, Qingdao, 266071 People’s Republic of China; 2 Laboratory for Marine Biology and Biotechnology, Qingdao National Laboratory for Marine Science and Technology, Qingdao, 266071 People’s Republic of China; 3 University of Chinese Academy of Sciences, Beijing, 100049 People’s Republic of China; 4 Ocean School, Yantai University, Yantai, 264005 People’s Republic of China; 5 Verschuren Centre for Sustainability in Energy and Environment, University of Cape Breton, Sydney, Nova Scotia Canada

**Keywords:** Seaweed, Dehydration, Ubiquitination, Phosphatidylinositol signaling system, Weighted gene co-expression network analysis

## Abstract

**Background:**

Intermittent dehydration caused by tidal changes is one of the most important abiotic factors that intertidal seaweeds must cope with in order to retain normal growth and reproduction. However, the underlying molecular mechanisms for the adaptation of red seaweeds to repeated dehydration-rehydration cycles remain poorly understood.

**Results:**

We chose the red seaweed *Gloiopeltis furcata* as a model and simulated natural tidal changes with two consecutive dehydration-rehydration cycles occurring over 24 h in order to gain insight into key molecular pathways and regulation of genes which are associated with dehydration tolerance. Transcription sequencing assembled 32,681 uni-genes (GC content = 55.32%), of which 12,813 were annotated. Weighted gene co-expression network analysis (WGCNA) divided all transcripts into 20 modules, with Coral2 identified as the key module anchoring dehydration-induced genes. Pathways enriched analysis indicated that the ubiquitin-mediated proteolysis pathway (UPP) and phosphatidylinositol (PI) signaling system were crucial for a successful response in *G. furcata*. Network-establishing and quantitative reverse transcription PCR (qRT-PCR) suggested that genes encoding ubiquitin-protein ligase E3 (E3–1), SUMO-activating enzyme sub-unit 2 (SAE2), calmodulin (CaM) and inositol-1,3,4-trisphosphate 5/6-kinase (ITPK) were the hub genes which responded positively to two successive dehydration treatments. Network-based interactions with hub genes indicated that transcription factor (e.g. TFIID), RNA modification (e.g. DEAH) and osmotic adjustment (e.g. MIP, ABC1, Bam1) were related to these two pathways.

**Conclusions:**

RNA sequencing-based evidence from *G. furcata* enriched the informational database for intertidal red seaweeds which face periodic dehydration stress during the low tide period. This provided insights into an increased understanding of how ubiquitin-mediated proteolysis and the phosphatidylinositol signaling system help seaweeds responding to dehydration-rehydration cycles.

## Background

As the boundary between land and sea, the intertidal zone has a unique ecological environment caused by the changing of tides and period of exposure due to the amplitude between spring and neap tides. Seaweeds growing in the intertidal zone are subject to sometimes extreme abiotic stresses during low tide, such as dehydration, strong solar irradiance and fluctuating temperature [1, 2]. Among all of these stressors, dehydration of the algal tissues is one of the most important limitations determining the upper and lower vertical distributions of intertidal seaweeds [3, 4]. Water loss can cause osmotic stress, mechanical damage to membrane systems and intra-cellular oxidative stresses which are induced by excessive reactive oxygen species (ROS) [2, 4–6]. In addition, dehydration can affect the main physiological and biochemical processes in seaweeds, including photosynthesis, protein synthesis and energy metabolism [4, 5, 7, 8]. Therefore, it is of great significance to study both the adaptive and tolerance mechanisms of seaweeds when responding to water deficit stress caused by exposure.

When facing desiccation stress, plant can usually activate rapid transduction of environmental stress signals and tolerance-related biochemical regulatory mechanisms [9]. Some studies on dehydration mechanisms in various seaweeds illustrated that increased antioxidant enzymes and components associated with detoxification were responsible for eliminating over-production of ROS [2, 4, 5, 10]. Meanwhile, the accumulation of compatible solutes can regulate cellular osmotic pressure and protect seaweed tissues during the dehydration process [11–13]. Other studies have paid much attention to the effect of dehydration on photosynthetic activity and cyclic electron flow [4, 8, 14, 15]. Nevertheless, some aspects relating to the actual mechanisms of dehydration tolerance are still unsolved. For example, the studies mentioned above did not uncover the associated upstream regulation and signal transduction systems. Recently, some studies illustrated that certain algae such as red seaweed *Pyropia orbicularis* and streptophyte green alga *Klebsormidium* share some common responding mechanisms with mosses or higher plants during water loss stress [8, 16, 17]. These findings inspired us to explore potential molecular pathways responsive for dehydration tolerance that have been previously overlooked in seaweeds. For example, the ubiquitin system is crucial for higher plants in their responses to drought stress [18, 19], but still poorly studied in seaweeds.

*Gloiopeltis furcata* (Rhodophyta) is a marine macroalga which occurs abundantly on the upper, rocky intertidal zone in the North Pacific coast [20, 21]. It has an important economic value in food, textile and traditional medicine [22]. Extracts of *G. furcata* have a variety of proposed functions such as cancer prevention and blood anti-coagulation [23–25]. Ecologically, seaweeds inhabiting the upper interidal zone (exposed for the greatest duration) are believed to have considerable potential to tolerate dehydration [3, 4]. In accordance with this expectation, *G. furcata* is indeed able to tolerate water loss for more than 72 h, despite nearly 80% of tissue water content being lost during low tide [26]. More specifically, even with a tissue water content as low as 6%, *G. furcata* could recover photosynthetic activity after complete submergence and rehydration [26]. Recently, *G. furcata* was reported to have a high O^2^-radical-scavenging activity [27]. These results indicated that *G. furcata* was an ideal model for studying dehydration-induced acclimation strategies of red seaweeds in intertidal ecosystems.

**Fig. 1.**
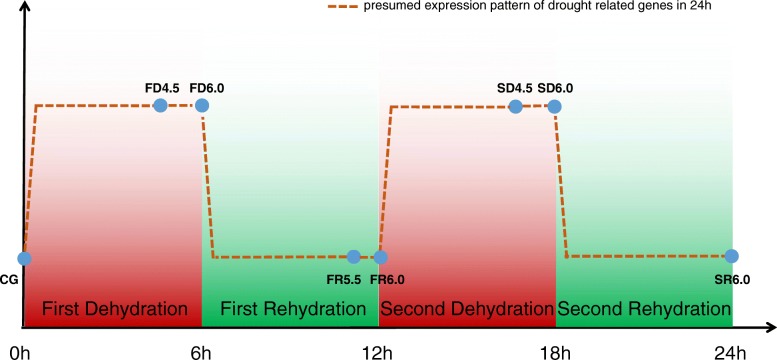
Diagram of experimental design and sampling points. 0-6 h was the first dehydration treatment (FD), 6 h-12 h was the first rehydration treatment (FR), 12 h-18 h was the second dehydration treatment (SD), 18 h-24 h was the second rehydration treatment (SR). All periods of dehydration stress are marked in red, while submerged (rehydration) periods are marked in green. The blue circles represent sampling time points, the orange dotted line represents the presumed expression pattern of dehydration-related genes

In this study, we chose *G. furcata* as research material and simulated two dehydration-rehydration cycles in the laboratory (Fig. 1), with goals to find the key pathways and genes related to dehydration stress by using RNA-sequencing and weighted gene co-expression network analysis (WGCNA)*.* Considering that natural tidal changes are continuously cyclic, continuous periodic treatments can help us to better understand the responding during dehydration stress. The results provide valuable insights for understanding the molecular mechanisms associated with dehydration of intertidal seaweeds.

## Results

### Overlook of transcriptome assembly

After assembly and filtration (see Materials and Methods), all clean reads were assembled into 32,681 uni-genes with an average length of 799 bp, and N50 was 1238 bp (Table 1). The average GC content of the uni-genes was 55.32% (Table 1). Twenty-four cDNA libraries were generated by RNA sequencing, and the number of reads (after filtration, see Materials and Methods) per library ranged from 21.90 M to 34.58 M (Additional file 1: Table S1). Statistical analyses of uni-genes expression levels in each sample showed that most of uni-genes were in the range of 0.5–5 RPKM (reads per kilobase of exon region per million mapable reads) and 5–100 RPKM. Approximately, 61% of expressed uni-genes had RPKM values ≤5, and 39% had RPKM values ≥5 (Additional file 2: Fig. S1). BLAST results indicated that 12,813 (39.2%) uni-genes were annotated into at least one of the following databases: the Kyoto Encyclopedia of Genes and Genomes database (KEGG) and Clusters of Orthologous Groups of proteins (KOG), NCBI non-redundant protein sequences (Nr) and Swissprot, 4815 uni-genes were annotated into all of these databases (Additional file 3: Fig. S2).

**Table 1:** Overview of *G. furcata* transcriptome information

Item	Number
Total assembled bases	26,117,344
Total uni-genes identified	32,681
GC %	55.32
N50 (bp)	1238
Max length (bp)	12,627
Min length (bp)	201
Average length (bp)	799

Fifteen uni-genes were selected for quantitative reverse transcription PCR (qRT-PCR) analysis in order to validate the quality of RNA-Seq data and 75% of which had a correlation coefficient ≥ 0.8, suggesting a strong consistency between quantitative results and transcriptome data (Additional file 4: Table S2). Such a correlation between RNA-Seq and qRT-PCR confirmed the high reliability of transcriptomic profiling data.

### Modules associated with dehydration tolerance by WGCNA

**Fig. 2.**
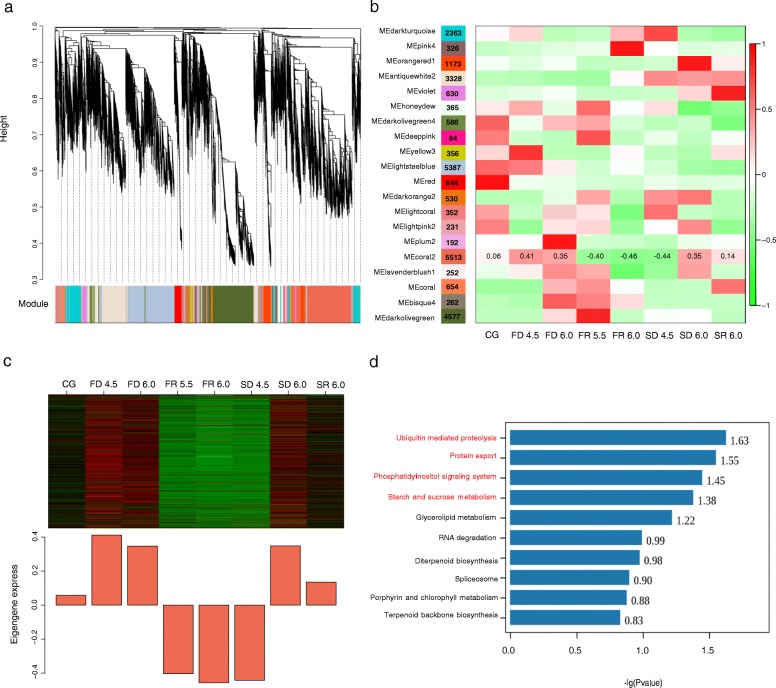
WGCNA of drought-associated genes in *G. furcata*. **a**: Clustering dendrogram and modules for 27805 uni-genes. Each gene is represented by a leaf in the tree, the y axis represents network distance, as determined by topological overlap (TO), different colors shows module membership after being merged. **b**: Module-trait relationships. Colors on the left represent twenty modules and the numbers of uni-genes were written in each module. A heat-map shows the module eigen-gene (ME) correlations to traits (eight samples for experiment). Numbers in the Coral2 analysis reported the correlation coefficients for the ME-trait relationship. **c**: Eigengene expression profile for Coral2 module. The bar-plot reported eigengene expression at each sampling point. For the heat-map, rows corresponded to genes, columns to samples, the green color represented under-expressed, the red denoted over-expressed. **d**: Top ten statistics of KEGG pathway enrichment. The names of those enriched pathways with *P*< 0.05 were marked in red

According to the filter standard (see Materials and Methods), 4876 uni-genes were removed. A topological overlap matrix (TOM) was generated using a set of 27,805 uni-genes for WGCNA (Additional file 5: Fig. S3). After a dynamic tree cut and merging, twenty modules were identified and designated by different colors, with gene numbers ranging from 84 (Deeppink) to 5513 (Coral2) (Fig. 2a and b). Module-trait relationships and eigengenes expressions of each module indicated that Coral2 was characterized by two clear fluctuations, in accordance with the two cyclic treatments of dehydration (Fig. 2b and c). Specifically, the expression patterns of genes grouped into Coral2 were up-regulated during two dehydration treatments, and down-regulated during rehydration (Fig. 2b and c). Although the gene expression at SD4.5 did not show significant increase, all other time points were in line with expectations (Fig. 2c). The Coral2 module was thus chosen for the following analysis.

### Pathway enrichment analysis of Coral2 provided insight into phosphatidylinositol signal transduction and ubiquitin mediated proteolysis

5513 uni-genes were grouped into the Coral2 module, and the functional pathways were characterized in Coral2 using GO analysis and KEGG enrichment study. A large number of uni-genes in Coral2 were involved in genetic and environmental information processing and metabolism. Statistics on GO terms of uni-genes in Coral2 showed that many uni-genes involved in cellular process and metabolic process, and the molecular function of binding and catalytic activity had the largest number of uni-genes (Additional file 6: Fig. S4). KEGG pathway enrichment analysis revealed that Coral2 was enriched (*P* < 0.05) in the ubiquitin mediated proteolysis pathway (UPP), protein export, phosphatidylinositol signaling system (PI signal system), and both starch and sucrose metabolism (Fig. 2c, Table 2, Additional file 7: Table S3). Many uni-genes belonging to the UPP and PI signals had a ≥ 1.5 fold change of RPKM value during drought stress, with very low expressions during rehydration (Table 2). We subsequently constructed co-expression networks to look for hub genes in these two pathways, considering that the function of UPP in dehydration response may have been neglected in seaweeds and the PI signal pathway still has not been studied clearly.

**Table 2:** Uni-genes annotated into ubiquitin-mediated proteolysis and phosphatidylinositol signaling system

Gene ID	Fold change								KME	Annotation
	CG	FD4.5	FD6.0	FR5.5	FR6.0	SD4.5	SD6.0	SR6.0		
Ubiquitin mediated proteolysis										
Gf07512	1.00	**1.62**	**1.68**	0.30	0.00	0.03	**1.68**	1.33	0.99	Transcription elongation factor B polypeptide 1
Gf18542	1.00	1.45	**1.60**	0.18	0.00	0.05	1.43	0.91	0.99	RING-box protein 1
Gf32620	1.00	1.24	0.99	0.00	0.00	0.00	1.24	0.96	0.97	Histone deacetylase complex subunit Cti6
Gf16090	1.00	**2.43**	**2.21**	0.14	0.00	0.00	**1.73**	1.09	0.97	E3 ubiquitin-protein ligase TRIP12
Gf27969	1.00	**1.76**	**1.70**	0.00	0.00	0.00	1.09	0.95	0.97	Ubiquitin-protein ligase E3
Gf18536	1.00	**1.62**	**1.50**	0.19	0.00	0.03	1.00	1.01	0.97	E3 ubiquitin-protein ligase UPL3
Gf08625	1.00	**3.30**	**3.00**	0.08	0.00	0.15	**2.84**	2.12	0.97	Elogin binding protein-like protein, partial
Gf19266	1.00	**1.84**	1.28	0.00	0.00	0.00	1.20	1.41	0.96	Unnamed protein product
Gf18480	1.00	**1.58**	**2.30**	0.00	0.00	0.00	**2.00**	1.23	0.96	TPR repeat-containing protein
Gf13903	1.00	1.33	1.02	0.48	0.40	0.45	1.21	0.76	0.95	Peptidyl-prolyl cis-trans isomerase-like 2
Gf19264	1.00	**2.90**	**1.74**	0.20	0.00	0.02	**1.70**	1.37	0.94	Unnamed protein product
Gf30775	1.00	**3.18**	**1.68**	0.05	0.00	0.06	**2.70**	1.61	0.94	ERAD-associated E3 ubiquitin-protein ligase
Gf14989	1.00	**1.92**	**1.81**	0.00	0.00	0.00	0.88	1.50	0.92	Unnamed protein product
Gf18742	1.00	0.97	1.45	0.00	0.00	0.07	**1.57**	1.43	0.92	Ubiquitin-activating enzyme E1
Gf19263	1.00	**2.34**	1.16	0.01	0.00	0.00	1.07	1.12	0.91	SUMO-activating enzyme subunit 2
Gf19265	1.00	**2.29**	0.74	0.00	0.00	0.00	1.25	1.03	0.87	SUMO-activating enzyme subunit 2
Gf11232	1.00	**1.50**	**2.06**	0.83	0.00	0.12	1.27	1.42	0.89	Ubiquitin-conjugating enzyme E2 34-like
Gf06247	1.00	**1.73**	**5.96**	0.11	0.00	0.00	**4.01**	4.29	0.78	E3 ubiquitin-protein ligase
Gf10562	1.00	**1.73**	1.45	0.46	0.32	0.40	0.56	1.69	0.75	Ubiquitin carrier protein
Phosphatidylinositol signaling system										
Gf03841	1.00	0.92	0.94	1.18	1.17	1.17	1.06	0.92	−0.87	IP_6_, PP-IP_5_ or IP_7_ kinase
Gf07443	1.00	**2.23**	**1.62**	0.00	0.00	0.01	**1.62**	1.82	0.96	Calmodulin
Gf08993	1.00	**2.27**	**3.21**	0.03	0.00	0.00	**2.39**	3.03	0.91	3′(2′),5′-bisphosphate nucleotidase, SAL3
Gf13982	1.00	1.22	0.99	0.55	0.81	0.79	1.02	0.71	0.74	Inositol-hexakisphosphate kinase
Gf14233	1.00	1.16	1.43	0.26	0.00	0.12	1.03	0.84	0.96	Phospholipase C (by KO-ID)
Gf18777	1.00	**4.85**	**1.84**	0.00	0.00	0.00	**1.83**	1.45	0.82	Inositol-1,3,4-trisphosphate 5/6-kinase
Gf30993	1.00	0.71	0.60	0.69	0.75	0.70	0.92	0.87	0.19	3 (2) -bisphosphate nucleotidase
Gf31439	1.00	1.44	1.08	0.69	0.17	0.33	0.95	0.21	0.72	Calmodulin 1

The expression level of the genes were shown as fold changes of RPKM comparing to CG. Any fold change higher than 1.5 in four dehydration treatments (FD 4.5, FD6.0, SD4.5, SD6.0) considered as significant increases and used bold type

### Identification of key genes related to UPP

**Fig. 3.**
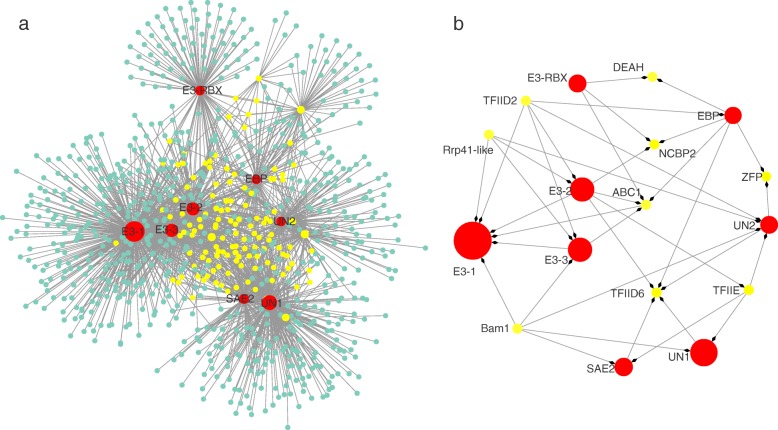
The Coral2 module-based ubiquitin-mediated proteolysis pathway (UPP) networks. **a:** The network for UPP in Coral2. This network was constructed by extracting Coral2 genes which annotated to UPP as seed nodes, with an edge weight cu-off of 0.45. Hub genes present in the network were coded red and those nodes with at least four neighbours within one distance were coded yellow. The node size represented the level of connectivity. **b**: The sub-network for hub genes in UPP and candidate hub-interacted genes. The sub-network was built by extracting the hub genes and the selected candidate co-expression genes connected to more than one hub genes within the UPP network

A UPP network was built by UPP-related genes and their co-expression genes in Coral2. The network contained 857 nodes and 1783 edges (Fig. 3a and Additional file 8: Table S4). Eight ubiquitin-related genes exhibiting high connectivity (> 100) were indicated in red, and four with interactions ≥150 were considered as large hubs (Fig. 3a). These large hubs were annotated as one unnamed protein product (*Gf19264*/UN1) and three E3 ubiquitin ligase (*Gf27969*/E3–1, *Gf16090*/E3–2, *Gf18536*/E3–3) (Fig. 3a and Additional file 8: Table S4). Four other mid-size hubs (100 < degree < 150) were *Gf19263* (SUMO-activating enzyme sub-unit 2/SAE2), *Gf19266* (unnamed protein product/UN2), *Gf08625* (elogin-binding protein-like protein/EBP) and *Gf18542* (RING-box protein 1/E3-RBX) (Fig. 3a). Amongst these mid-size hubs, SAE2 was related to the small ubiquitin-related modifier (SUMO) conjunction. E3-RBX was also one kind of E3 with a RING-box domain. Thus, four of the eight hubs in the UPP network encoded for different E3 ligases and interacted with 589 nodes (Fig. 3a).

Nodes with at least four neighbors within one distance were extracted, in order to investigate if there was a common up-stream regulator or downstream substrate for the hub genes in UPP. In addition to hub genes, 128 nodes were selected and colored yellow (Fig. 3a). We focused on nine genes according to their descriptions, and extracted them with the hub genes to built a fine-scale sub-network. (Fig. 3b and Additional file 8: Table S4). We found seven genes involved in genetic information processing, two genes encoded for sub-units 2 and 6 of the transcription factor TFIID (*Gf03506*/TFIID2, *Gf26104*/TFIID6); one gene encoded the alpha sub-unit of transcription initiation factor IIE (*Gf17133*/TFIIE by KO ID) and one gene encoded for the zinc-finger domain containing protein (*Gf31441*/ZFP) (Fig. 3b and Table 3); three othergenes were involved in RNA processing, such as *Gf06149* (exosome component 4-like /Rrp41-like), *Gf30426* (nuclear cap-binding protein sub-unit 2/NCBP2), *Gf32444* (DEAH-box RNA helicase/DEAH) (Fig. 3b and Table 3). Moreover, *Gf24243* (kin (ABC1)/ABC1) and *Gf12536* (Beta amylase/Bam1) relating to osmotic regulation also interacted with hub genes in UPP (Fig. 3b and Table 3).

**Table 3:** Candidate hub-interacted uni-genes responsive to desiccation in the UPP and PI signal sub-network

Genes	Network	Description	KEGG pathway
Genes participate in genetic information processing			
Gf06149	CN	Exosome component 4-like Rrp41-like	ko03018//RNA degradation
Gf03506	CN	Transcription factor TFIID sub-unit D2	ko03022//Basal transcription factors
Gf32444	CN	DEAH-box RNA helicase	–
Gf26104	UPP	Transcription factor TFIID sub-unit 6	ko03022//Basal transcription factors
Gf30426	UPP	Nuclear cap-binding protein sub-unit 2	ko03040//Spliceosome; ko03013//RNA transport
Gf31441	UPP	Zinc-finger domain containing protein	–
Gf17133	UPP	Transcription factor TFIIE (by KO-ID)	ko03022//Basal transcription factors
Genes related to osmotic regulate			
Gf12536	CN	Beta amylase, Bam1	ko00500//Starch and sucrose metabolism
Gf24243	CN	Kin (ABC1)	–
Gf25392	PI	Major intrinsic protein	–

CN was the abbreviation of common hub-interacted uni-genes in two sub-networks

### Identification of key genes related to PI signal transduction

**Fig. 4.**
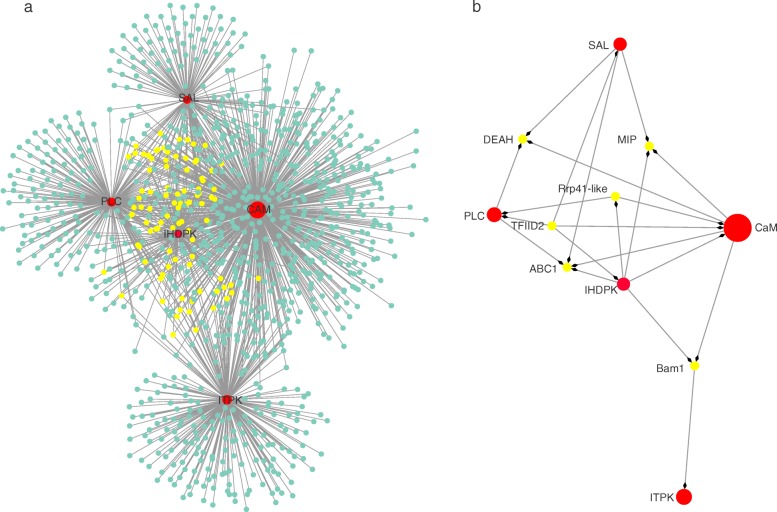
The Coral2 module-based phosphatidylinositol (PI) signaling system networks. **a:** The network for PI signal system. This network was constructed by extracting those Coral2 genes which annotated to the PI signal pathway as seed nodes, with an edge weight cut-off of 0.4. The hub genes present in the network were coded red and the nodes with at least three neighbours within one distance were coded yellow. The node size represents the level of connectivity. **b**: The sub-netwok for hub genes in PI signal and candidate hub-interacted genes. The sub-network was built by extracting hub genes in the UPP and the selected candidate co-expression genes connected to more than one hub genes

The Coral2 module-based co-expression network for PI signal transduction indicated that the PI signal network contained 1029 nodes and 1433 edges (Fig. 4a and Additional file 9: Table S5). Four large hubs such as *Gf07443* (calmodulin/CaM), *Gf18777* (inositol-1,3,4-trisphosphate 5/6-kinase/ITPK), *Gf08993* (3′(2′), 5′-bisphosphate nucleotidase/SAL) and *Gf14233* (phospholipase C/PLC by KO ID) were identified in the PI network (Fig. 4a). One mid-size hub was *Gf03841*(inositol hexakisphosphate (IP_6_) and diphosphoinositol-pentakisphosphate (PP-IP_5_ or IP_7_) kinase/IHDPK)(Fig. 4a). Among these hubs, CaM, served as a calcium receptor protein, which had the highest connectivity (Fig. 4a). ITPK and SAL not only participated in the phosphatidylinositol signaling system but also in inositol phosphate metabolism (Additional file 9: Table S5).

Ninety-three nodes (except hub genes) with at least three neighbors within one distance were colored yellow in order to find the potentially common interacted genes with hubs (Fig. 4a). Six of them were chosen to build the sub-network (Fig. 4b and Additional file 9: Table S5). Surprisingly, five of these also had co-expression with hub genes in the UPP network, such as Rrp41-like, DEAH, ABC1, Bam1 and TFIID2 (Figs. 3b and 4b and Table 3). Bam1 was also considered to play a key role in the pathway of starch and sucrose metabolism which was enriched in Coral2 (Additional file 7: Table S3). Gf25392 (major intrinsic protein /MIP), one kind of aquaporin (AQP) that regulates cellular water balance under desiccation conditions, was also found to have some interactions with the PI network hub genes (Fig. 4b).

### Expression patterns of four candidate drought-related hub genes

**Fig. 5.**
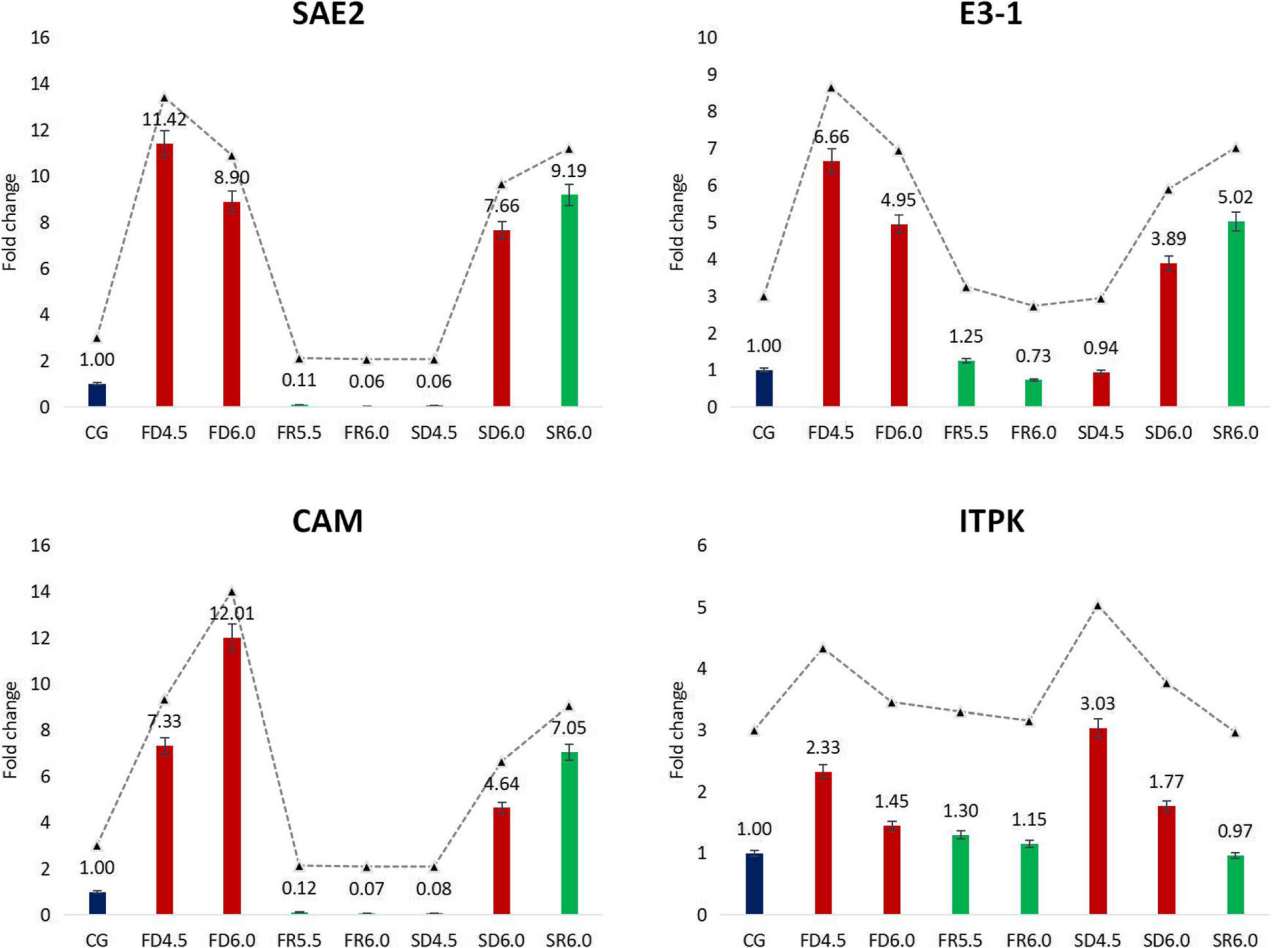
qRT-PCR analysis of expression levels of four candidate dehydration-related hub genes in both UPP and PI signal networks. Columns represented the fold-change values of each sample, the broken line represented the trend of genes expression. Each of them had three technical replicates

Combining the fold changes of the RPKM value, the result of co-expression networks and the annotations of genes, we chose E3–1, SAE2, CaM, ITPK as candidate dehydration-responsive genes, and identified their transcriptional expression with qRT-PCR. As shown in Fig. 5, expression patterns of four hub genes demonstrated their positive responses to dehydration stress. CaM, E3–1 and SAE2 genes all showed increases during first dehydration and was down-regulated when submerged (Fig. 5). The expression patterns of the second simulated low tide cycle were not exactly the same as the first. Specifically, the expression levels of CaM, E3–1 and SAE2 genes increased at FD4.5 during the first cycle, but began to rise at SD6.0 during the second cycle, and the fold changes at SD6.0 were lower than at FD4.5 or FD6.0 (Fig. 5). These three genes were all down-regulated during first rehydration, especially SAE2 and CaM almost reduced to zero at FD4.5 and FD6.0. However, they did not show downtrends at SR6.0. ITPK was up-regulated at four time points during dehydration treatment, peaked at FD4.5 and SD4.5, and reduced to almost normal level after rehydration (Fig. 5).

## Discussion

### Post-translation modifications mediated dehydration response in *G. furcata*, E3 and SAE2 were the key genes induced during exposure to dehydration

As an important regulatory mechanism of post-translation modifications, ubiquitination has been widely recognized as a mechanism to explain how higher plants respond to drought stress [18, 19, 28, 29]. In this study, UPP was significantly enriched in the dehydration-related Coral2 module, the transcript level of many genes in UPP showed up-regulation in at least one dehydration treatment group and were significantly down-regulated during dehydration (Fig. 2d and Table 2). We therefore suggest that UPP may have played an important role in the dehydration-response of *G. furcata*.

Growing evidence indicates that UPP has a functional role of tolerating abiotic stress in those seaweeds studied this far, despite most of these studies focusing on heat shock stress [30–33]. A study of the red alga *Gracilaria lemaneiformis* demonstrated that the bioactivity of UPP was directly related to its ability to withstand heat stress [33]. Nevertheless, research linking UPP to dehydration in macroalgae are still very scarce. One study of dehydration-tolerant seaweed *Fucus vesiculosus* reported on the over-expression of two ubiquitin-ribosomal protein fusion genes, thereby suggesting that the protein targeting and degradation pathway, via 26S proteasome, was up-regulated during dehydration [34]. Another study of the high intertidal red seaweed, *P. orbicularis* showed a significant increase of the transcript level of ubiquitin during water loss and down regulation during rehydration [5]. The results presented here on the intertidal red alga *G. furcata* further support the viewpoint that, as with higher plants, ubiquitin production is also a crucial mechanism for exposed seaweeds to adapt to periodic dehydration stress during low tides.

Ubiquitin-activating enzymes (E1), ubiquitin-conjugating enzymes (E2) and ubiquitin ligase enzymes (E3), and their concerted actions are required for UPP [35, 36]. In the dehydration-related module Coral2, six uni-genes encoded different E3 and four of them were found as the hubs in the UPP network (Table 2 and Fig. 3a). This was taken to indicate that diverse kinds of E3 exist in *G. furcata* and likely play vital functions in UPP. In fact, E3 are the most diverse enzymes in the ubiquitin proteasome system and have been divided into many different types based on sub-unit compositions (e.g. HECT type, F-box type, RING type, U-box type) [36]. E3 determine the specific selection and recognition of substrate proteins in UPP [35, 37], the fact that E3 interact with different targets accounts for making them important hubs in UPP network.

In *G. furcata*, we found one candidate dehydration-induced E3 (E3–1) significantly responded to water loss because its expression increased markedly at FD4.5, FD6.0 and SD6.0 (Fig. 5). We propose that E3 positively responding to water deficit and resisted the negative influences caused by exposure to dehydration. In fact, many types of E3 have been identified in higher plants to participate in tolerating drought stress by reducing oxidative stress and regulating downstream genes (e.g. drought-related transcription factors) [38–41]. Our study is the first report of an E3 expression profile significantly associated with a dehydration response in macroalgae. Similarly, differential expression analysis in the red intertidal macroalga, *Pyropia haitanensis* showed that long-term exposure to high temperature, another major abiotic stress for intertidal seaweeds, also induced the expression of E3 [42].

In addition to E3, another hub gene SAE2 related to SUMOylation also showed high connectivity with other genes in the UPP network, with an almost 7–10 fold increment at different time points of the dehydration treatment (Table 2 and Fig. 5). Such a pattern enabled us to argue that SAE2 also played an important role in dealing with the stresses of water loss. SAE2 is an essential large sub-unit for the SUMO-activating enzyme (SAE) in SUMOlaytion [43].SUMOlaytion is another important form of post-translational modification similar to UPP, which also plays crucial roles in regulating what when responding to abiotic stress responses, particularly in plant drought stress [44–46]. Some studies suggest that SAE may act as a limiting regulatory step during SUMO conjugation [43, 47]. It was found that when SUMO conjugation was impaired by the expression of the SAE2^UFDCt^ domain, plants became more sensitive to drought [48].

### PI signal connected with Ca^2+^/CaM pathway responded to dehydration, and the role of CaM and ITPK in signal transduction

When land plants are faced with abiotic stresses, timely signal transmission can activate response mechanisms which enable them to respond and resist [49, 50]. The PI signal system, including a series of kinases and phosphatases, is involved in the perception and transduction of external stimuli [49, 51]. In this study, the PI signal pathway showed a significant enrichment in the dehydration-related module, supporting its crucial function during water loss in *G. furcata* (Fig. 2d).

The PI signal also interacted with the Ca^2+^/CaM signal pathway [49, 51, 52]. Here, CaM is an essential part of the Ca^2+^/CaM signal [53], and showed the highest connectivity in the PI signal network, its expression was affected by water loss during both of the periodic dehydration treatments (Figs. 4a and 5). These results suggested that for intertidal seaweeds, the PI signal pathway most likely interacts with Ca^2+^/CaM signals in order to construct a complex signal regulatory network for transducing external stress signals and activating downstream pathways in response to dehydration.

CaM is an ubiquitous Ca^2+^-binding protein related to many biochemical reactions, and can be activated by Ca^2+^ release in response to multiple environmental stimuli [53]. The intertidal red seaweed *Porphyra yezoensis* also increased the expression level of the CaM gene when loss of tissue water reached 20%, the expression peaked when reaching 40% [54]. Likewise, CaM showed an increased transcription level in *P. orbicularis* during dehydration and returned to the normal levels when rehydrated [5]. This environment-induced change of CaM has also been reported under copper and temperature stresses in other seaweeds [55, 56].

Although the PI signal pathway is associated with the Ca^2+^/CaM signal pathway in stress-induced signal networks, how they interact with each other is still largely unknown. One connection between these two pathways is through the secondary messenger inositol 1,4,5-trisphosphate [Ins (1,4,5) P_3_], generated by PLC hydrolysis [49, 51, 57, 58]. Ins (1,4,5) P_3_ can release Ca^2+^ from the endoplasmic reticulum (ER) to change the concentration of Ca^2+^ and regulate downstream mechanisms with activated CaM [49, 51, 57]. In turn, the activities of PLC can be stimulated by higher Ca^2+^ concentration [58, 59]. Our results from *G. furcata*, however, do not seem to support this viewpoint. Although one hub gene (*Gf14233*) in the PI network annotated as PLC by KO-ID, but the expression level of PLC was not affected significantly by water loss (Fig. 4a, Table 2). Actually, genes encoding for the Ins (1,4,5) P_3_ receptor have not been found in plants [60]. More importantly, the pleckstrin homology (PH) domain and EF hand, the important structural domains for membrane binding and Ca^2+^-dependent activation of PLC, were reported to be absent in red seaweeds [60].

Another drought-induced hub gene ITPK in the PI signal network (Figs.4a and 5) allows us to propose that the PI signal may connect with the Ca^2+^/CaM pathway by other inositol phosphates in *G. furcata*. ITPK is the key regulatory enzyme at the branch point for the synthesis of InsP_4_ isomers [e.g.Ins (1,3,4,5) P_4_, Ins (1,3,4,6) P_4_ and Ins (3,4,5,6) P_4_], inositol pentakisphosphate (InsP_5_) and inositol hexaphosphate (InsP_6_) [61–64]. Recent studies showed that inositol metabolites such as InsP_6_ and Ins (1,3,4,6) P_4_ may also alter cytosol Ca^2+^concentration under stress conditions [49, 62, 63]. ITPK can also inter-convert Ins (3,4,5,6) P_4_ and Ins (1,3,4,5,6) P_5_ within a substrate cycle to regulate the concentration of Ins (3,4,5,6) P_4_ [64–66]. Ins (3,4,5,6) P_4_ can act as the second messenger [67], and play important roles in signal transduction [67–69]. In this study, the dehydration-induced transcript levels of ITPK indicated it was associated with dehydration stress (Fig. 5) by impacting signal molecules, as reported in many higher plants [62, 63]. For *G. furcata*, CaM and ITPK may be at the key position responding to dehydration in terms of a signal transduction network. However, further work is need to verify the potential link between the PI and Ca^2+^/CaM signals by inositol phosphate such as InsP_4_ and InsP_6._

### UPP and PI signal co-expression with transcription factor, RNA modification and osmotic regulation related genes

The PI signal pathway and production of ubiquitin are up-stream regulator systems which can affect many mechanisms dealing with exposure to abiotic stresses [18, 49]. In *G. furcata*, we found that many hub genes in these two pathways co-expressed with transcription factors (e.g. sub-units 2 and 6 of TFIID2, TFIIE), RNA modification (e.g. Rrp41-like, DEAH, ZFP) and osmotic regulation-related genes (e.g. ABC1, Bam1, MIP) (Fig. 3b and 4b and Table 3). Other studies regarding drought tolerance in higher plants also showed that E3 ligase and CaM could regulate drought stress by interacting with transcription factor and other drought-resistant genes [70–73], providing much support to our results.

The candidate hub-related genes found in our study were mostly elevated to more than1.5 fold compare to CG at FD 4.5 and FD 6.0, and they exhibited obvious up-regulated trends at SD6.0 compared to FR6.0 (Additional file 10: Fig. S5). Some of them (i.e. DEAH, TFIID, ABC1, Bam1 and MIP) were reported to link to drought or other abiotic stresses. For example, DEAH was reported to be involved dealing with salt and Cd [74, 75]. ABC1 transgenic *Arabidopsis thaliana* showed an enhanced osmotic regulation ability [76], and ABC1 is also responsible for oxidative stress [77, 78]. TFIID was proposed a candidate gene for drought response and heat stress in higher plants [79–81], and Bam1 has been reported to participate in drought tolerance by degrading transitory starch to sustain proline [82, 83]. In addition, MIP in the PI signal network encoded for an aquaporin (AQP) (Fig. 4a), which can maintain cellular water balance under drought conditions and be regulated by a calcium signal [84]. These reports, along with the results from *G. furcata*, indicated that the ubiquitin mechanism and signal transduction system, including the PI and Ca^2+^ signals, may influence these drought-related regulator (e.g. TFIID) and osmotic defense genes (e.g. Bam1, MIP) in order to tolerate dehydration.

## Conclusions

The PI signal pathway, connected with the Ca^2+^/CaM signal, is required in order for *G. furcata*to transduction the external dehydration signal, whilst the ubiquitin-related pathway provided the post-translation modifications required to cope with dehydration stress in this intertidal red alga. These two pathways served as regulatory mechanisms which may interact with some dehydration-related transcriptional factor, RNA modification, and osmotic regulation genes thereby enabling this seaweed to tolerate considerable water loss at low tide. In these two pathways, E3, SAE2, CaM and ITPK were possibly at the key positions and induced significantly by dehydration. Further research should be conducted related to the complex functions of the ubiquitin mechanism operating in more seaweed species and to provide greater insight into the specific mechanisms of interactions between the PI and Ca^2+^/CaM signals.

## Materials and methods

### Plant materials and cultivation conditions

Specimens of the intertidal red alga *G. furcata* were collected at April from the upper, rocky intertidal zone in Yantai, Shandong, China (37°27′45.23″N, 121°26′34.28″E). We plucked the samples when *G. furcata* expose to the air during the low tide and all samples were transported to the laboratory with ice. After cleaning the surface with filtered seawater, we selected young and healthy thalli with uniform size and thickness (3–4 months young thalli about 3 cm long and 1.5 cm wide) for experiments. Before the formal treatment, according to previous study in *G. furcata* [26], our samples were pre-cultured at 11 °C with 50 μmol photons m^− 2^ s^− 1^ irradiation provided by cool-white fluorescent lamps in a 12:12 (light:dark) cycle.

### Experimental design and sampling

Considering the semi-diurnal tide in Yantai [85], and the physiological data reported in previous studies [86], we designed two continuous dehydration-rehydration cycles within 24 h to simulate the natural tidal cycle (Fig. 1). We dried the surface moisture of samples with filter papers and fixed the bottom of the thalli with wooden frame, then suspended individually in a ventilated culture box (GZP-250 N, Shanghai Senxin Co., Ltd., China) for 6 h as the first dehydration treatment (FD). Samples were taken out at 4.5 h and 6 h respectively during FD and immediately frozen in liquid nitrogen and stored at − 80 °C. After FD, the first rehydration treatment (FR) was continued, samples were submerged in the filtered sea water that previously placed at the culture box, and samples were taken out at 5.5 h and 6 h successively during FR. During the formal experiment, the temperature was controlled at 11 °C and the relative humidity was 77%. The treatments during the second dehydration (SD) and second rehydration (SR) were same as FD and FR mentioned above, and samples were taken out at 4.5 and 6 h during SD and 6 h during SR. The *G. furcata* were sampled at eight different time points during two dehydration-rehydration cycles, for convenience, the eight time points were abbreviated as CG (control group), FD4.5, FD6.0, FR5.5, FR6.0, SD4.5, SD6.0, SR6.0. At each time point we prepared more than three biological replications.

### RNA extraction, sequencing and transcriptome assembly

Total RNA was extracted from the thalli using the Plant RNA Kit 150 (Omega, USA) according to the manufacturer’s instructions. RNA degradation and contamination were monitored on 1% agarose gels and Nano-Photometer spectrophotometer (DeNovix, USA). As described above, we designed eight time points for RNA sequencing, and each had three biological replications. A total of 24 Seq-libraries were sequenced on Illumina HiSeq2000 instrument (Guangzhou Gene Denovo Biotechnology Co., Ltd., China). The raw reads were filtered by removing reads with adapters, reads with a ratio of N (the percentage of nucleotides in the reads that could not be sequenced) > 10% and low-quality reads (those reads containing over 40% bases with *Q* value < 20%), reads that mapped toribosome (rRNA) were also removed. For normalization of the data, gene expression levels were measured by the number of uniquely mapped reads per kilobase of exon region per million mapable reads (RPKMs).

### WGCNA analysis and network construction

For WGCNA (co-expression network analysis), uni-genes obtained from RNA-sequencing were filtered for a second time. Genes with RPKM < 1 in all samples or the coefficient of variation [(SD/Mean)*100%] < 0.1 were removed. All the remaining uni-genes were used for WGCNA with R-package [87]. According to the correlations between genes, the co-expression adjacency matrix was formed and converted to a topological overlap matrix (TOM). Co-expression modules were generated by hierarchical clustering and a dynamic tree cut, the minimum module size was set as 50 and modules with a tree height < 0.3 were merged together. The expression patterns of 20 modules were displayed as the eigenvalues (equivalent to the weighted synthesis values of all genes in each module and can reflect the comprehensive expression level for the module) [88]. The Coral2 module-based networks for UPP and PI signal pathways were constructed using genes annotated into these pathways as nodes to extract the co-expressed gene pairs. The resulting networks, with an edge weight cut off of 0.45 (for PI signal network) or 0.4 (for UPP network), were visualized by Cytoscape [89]. The hub genes are those showing the most connections in the network and often play important roles. In our study, genes which had degree values between 100 and 150 were considered as mid-size hubs, those genes with degree values > 150 were considered as large hubs.

### Gene annotation and pathway enrichment analysis

Description of uni-genes and pathway annotation were performed by BLASTing databases, including the Kyoto Encyclopedia of Genes and Genomes database (KEGG, http://www.genome.jp/kegg), Clusters of Orthologous Groups of proteins (KOG, http://www.ncbi.nlm.nih.gov/COG/KOG), NCBI non-redundant protein sequences (Nr, http://www.ncbi.nlm.nih.gov) and Swissprot (http://www.expasy.ch/sprot). In addition, GO analyse and KEGG enrichment test were also used to detect potential dehydration responsive function between co-clustered genes in the dehydration-related module. Pathways with *P* value < 0.05 were considered as significant enrichment.

### Gene expression analysis by qRT-PCR

RNA used for qRT-PCR was the same as that for previous transcriptome sequencing. For the first-strand cDNA synthesis, the PrimeScript™ RT Regen Kit with gDNA Eraser Kit (Takara, Kyoto, Japan) was used following the manufacturer’s instructions. The expression levels of selected genes were verified by qRT-PCR with the following cycling conditions: 95 °C for 30 s, followed by 40 cycles of 95 °C for 10 s, 55 °C for 10 s and 72 °C for 20 s. The qRT-PCR was conducted using SYBR® Premix Ex *Taq*™ II (Tli RNaseH Plus, Takara, Kyoto, Japan). The reactions were performed in 20 μL volumes containing 10 μL of 2 × SYBR® Premix Ex *Taq*, 0.8 μL of each primer (10 μM concentration of each primer), 3.0 μL of the diluted cDNA mix, and 6.4 μL of RNA-free water. A melting curve for each amplicon was then analyzed to verify the specificity of each amplification reaction. No template controls were included for each primer pair and each PCR reaction was carried out in three biological replicates. Elongation factor 2 (EF2) were used as internal controls [90, 91], and the sequence of EF2 was obtained in NCBI (GenBank: EF033553.1). The 2^-△△Ct^ method was used to calculate relative gene expression values. The sequences of the primers used are given in Additional file 11: Table S6, the primers concentrations were 10 μM.

## Supplementary information

**Additional file 1: Table S1.** The number of reads and identified uni-genes in each sample.

**Additional file 2: Fig. S1.** Statistics for the number of genes with different RPKM values in each library.

**Additional file 3: Fig. S2.** Venn diagram of the annotation of uni-genes in four databases.

**Additional file 4: Table S2.** The qRT-PCR results of uni-genes for testing transcriptome data.

**Additional file 5: Fig. S3.** Hierarchical clustering of the topological overlap matrix (TOM) of genes.

**Additional file 6: Fig. S4.** Summary of GO terms in Coral 2.

**Additional file 7: Table S3.** Table of Coral2 uni-genes annotated to KEGG pathway of protein export, starch and sucrose metabolism.

**Additional file 8: Table S4.** Table of all nodes in the UPP network.

**Additional file 9: Table S5.** Table of all nodes in the PI signal network.

**Additional file 10: Fig. S5.** Broken line chart of the candidate hub-interacted genes.

**Additional file 11: Table S6.** Table of primer sequence used in this study.

## Data Availability

All datasets generated or analyzed during this study are available from the corresponding author on reasonable request.

## Abbreviations

AQP: aquaporin
E3: Ubiquitin ligase E3
FD: First dehydration treatment
FR: First rehydration treatment
GO: Gene ontology
KEGG: Kyoto encyclopedia of genes and genomes
kME: Eigengene-based connectivity
PI: Phosphatidylinositol
qRT-PCR: quantitative reverse transcription PCR
ROS: Reactive oxygen species
RPKM: reads per kilobase of exon region per million mapped reads
SD: Second dehydration treatment
SR: Second rehydration treatment
SUMO: Small ubiquitin-related modifier
TOM: Topological overlap matrix
UPP: Ubiquitin-mediated proteasome pathway
WGCNA: co-expression network analysis
