# Supplementary figures and images for: PI signal transduction and ubiquitination respond to dehydration stress in the red seaweed Gloiopeltis furcata under successive tidal cycles
Source: BMC Plant Biol. 2019 Nov 27;19:516. doi: 10.1186/s12870-019-2125-z (PMC6880600; doi:10.1186/s12870-019-2125-z)

Figure S2


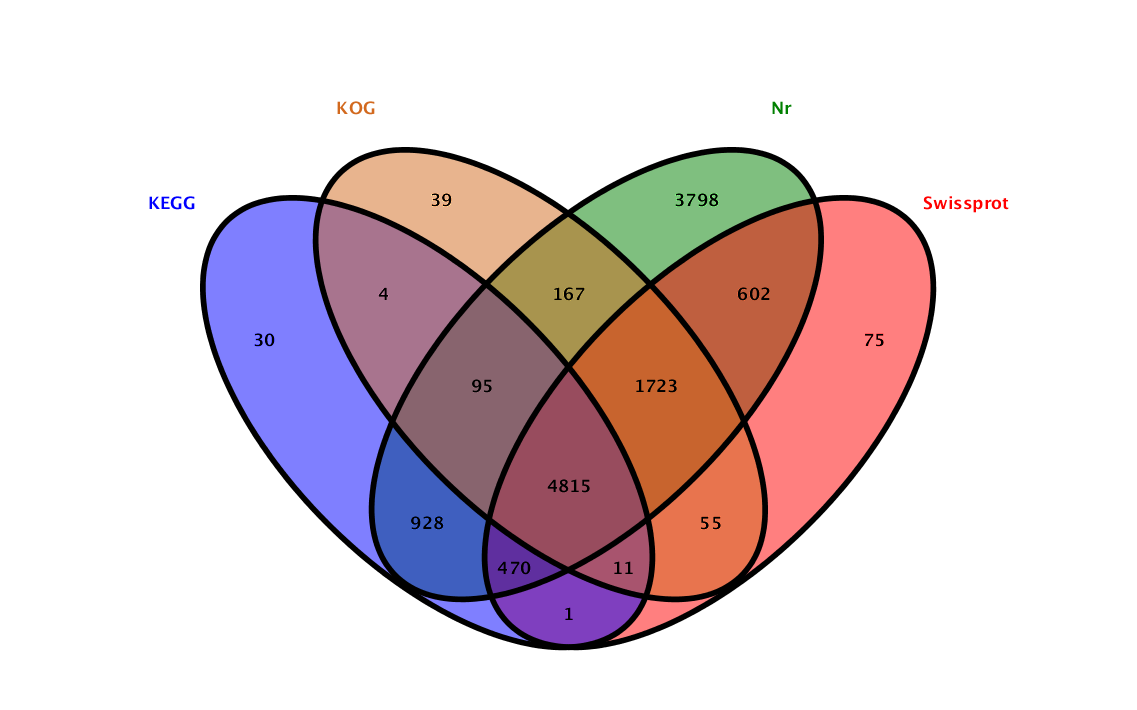

Supplement: Supplementary file 3 — Additional file 3: Fig. S2. Venn diagram of the annotation of uni-genes in four databases. [file 12870_2019_2125_MOESM3_ESM.docx]

Figure S3


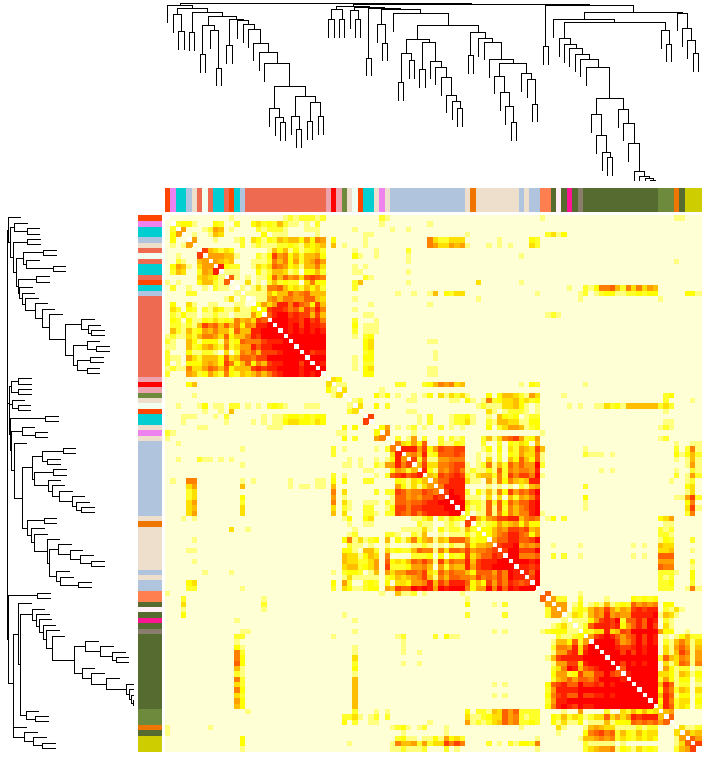

Supplement: Supplementary file 5 — Additional file 5: Fig. S3. Hierarchical clustering of the topological overlap matrix (TOM) of genes. [file 12870_2019_2125_MOESM5_ESM.docx]

Figure S4


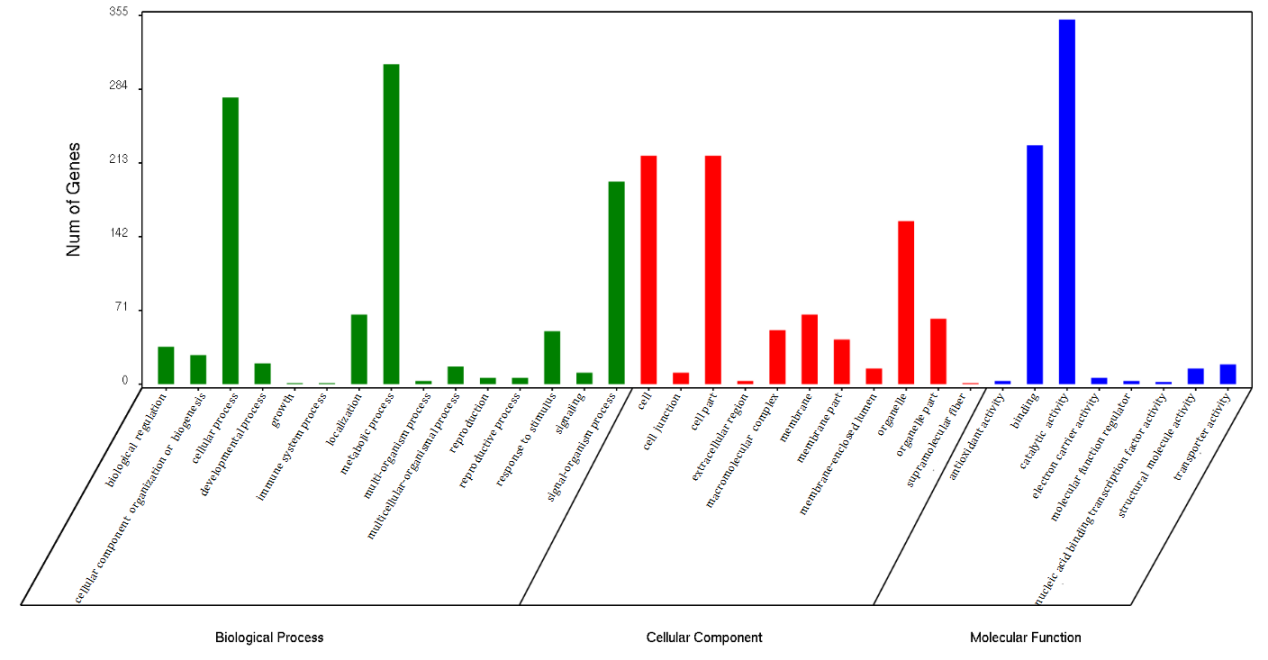

Supplement: Supplementary file 6 — Additional file 6: Fig. S4. Summary of GO terms in Coral 2. [file 12870_2019_2125_MOESM6_ESM.docx]
